# Initial β-hCG levels and 2-day-later increase rates effectively predict pregnancy outcomes in single blastocyst transfer in frozen-thawed or fresh cycles: A retrospective cohort study

**DOI:** 10.1097/MD.0000000000035605

**Published:** 2023-10-20

**Authors:** Gonul Ozer

**Affiliations:** a Memorial Sisli Hospital, IVF and Reproductive Genetics Centre, Istanbul, Turkey.

**Keywords:** biochemical pregnancy loss, clinical pregnancy loss, in vitro fertilization, live birth, β-hCG level

## Abstract

To investigate whether the initial beta-human chorionic gonadotropin (β-hCG) levels and their rate of increase differ after single fresh and frozen blastocyst transfers, and whether these values effectively predict pregnancy outcomes. This retrospective cohort study was conducted at the Sisli Memorial Hospital, assisted reproductive technology, and Reproductive Genetics Center in Istanbul, Turkey, between January 2016 and January 2022. Three thousand two hundred thirty-eight single blastocyst transfers with positive pregnancy test results were evaluated. Of these, 738 were fresh transfer cycles and 2500 were frozen-thawed embryo transfer (FET) cycles. β-hCG test results from 9 days after fresh and FET cycles were compared between the groups with biochemical pregnancy, early pregnancy loss, and live birth outcomes. The threshold values were determined for each pregnancy outcome. The rate of increase between the first and second β-hCG tests performed 2 days apart was determined for each pregnancy outcome. Finally, the listed values were compared between the FET and fresh cycle. Mean baseline β-hCG levels were significantly higher in FET cycles than in fresh cycles, regardless of pregnancy outcomes (*P* < .005). Baseline β-hCG levels were higher in fresh cycles with live births (171.76 ± 109.64 IU/L) compared to biochemical and clinical pregnancy losses (50.37 ± 24.31 and 114.86 ± 72.42, respectively) (*P* < .001). Live births in FET cycles resulted in higher baseline β-hCG levels (193.57 ± 100.38 IU/L) compared to biochemical and clinical pregnancy loss groups (68.41 ± 51.85 and 149.29 ± 96.99 IU/L, respectively) (*P* < .001). The β-hCG threshold for live birth for fresh cycles was 116.5 IU/L (sensitivity 80%, specificity 70%, positive predictive value 90%, negative predictive value 54%) and 131.5 IU/L for FET cycles (sensitivity 71%, specificity 68%, positive predictive value 87%, negative predictive value 50%). The percentage of the area under the curve for single fresh blastocyst transfers was 0.81 and 0.76 for frozen transfers. The rate of increase in β-hCG was similar in fresh and FET cycles. Initial β-hCG levels and 2-day increases are effective parameters for diagnosing pregnancy in fresh and FET cycles. The initial β-hCG level was significantly higher in the FET cycles than in the fresh cycles. Predicting outcomes earlier helps clinicians to manage and follow high-risk pregnancies.

## 1. Introduction

Couples undergoing assisted reproductive technology (ART) cycles experience significant stress and anxiety, especially during the initial pregnancy test.^[1,2]^ Early accurate predictors of pregnancy outcomes could potentially reduce the uncertainty and stress associated with ART.

Several serum biomarkers, including progesterone, estradiol, cancer antigen 125, activin, inhibin, pregnancy-associated protein-A, hyperglycosylated human chorionic gonadotropin (hCG), and beta-human chorionic gonadotropin (β-hCG), have been investigated to predict pregnancy outcomes.^[3]^ Serum β-hCG level is a commonly used marker for detecting pregnancy in vitro fertilization (IVF) centers. hCG is a glycoprotein hormone produced by syncytiotrophoblast cells of the placenta. It is composed of 2 non-covalently linked subunits: the alpha and beta subunits. The β-subunit is responsible for the biological activity of hCG.^[4]^ Its serum levels dynamically increase and double every 48 hours in most pregnancies, and this pattern is similar in both in vivo and in vitro conceptions.^[5]^ In ART cycles, serum β-hCG concentration should not be measured until 9 to 12 days after blastocyst transfer or 12 to 14 days after cleavage-stage embryo transfer because it has the potential for false-positive results due to residual β-hCG from the injections used to induce oocyte maturation.^[6]^ Several studies have reported various initial β-hCG threshold levels to explore the relationship between serum β-hCG levels and pregnancy outcomes.^[7–11]^ However, a few of these studies have compared the β-hCG thresholds for predicting clinical outcomes in pregnancies conceived by the transfer of single fresh and frozen-thawed blastocyst-stage embryos and have revealed conflicting results.

To date, there is no established threshold β-hCG level to assess pregnancy outcomes after a single fresh and frozen-thawed blastocyst-stage transfer. To address this gap, this study aimed to investigate the initial rate of increase in serum β-hCG levels following single fresh and frozen-thawed blastocyst-stage transfer and establish β-hCG thresholds for the prediction of biochemical pregnancy loss, early pregnancy loss, and live birth using receiver operating characteristic analysis.

## 2. Material methods

### 2.1. Study design and population

We performed a retrospective cohort study at the Sisli Memorial Hospital, ART and Reproductive Genetics Center in Istanbul, Turkey, including 2500 frozen-thawed and 738 fresh, single blastocyst transfers between January 2016 and January 2022. We retrospectively analyzed the electronic medical records of women who conceived with ART during the study period. The only cycles included in the study were fresh or frozen-thawed single blastocyst transfer cycles, and those in which the initial positive serum β-hCG level was detected on day 9 after transfer. The exclusion criteria were cycles in which cleavage-stage embryos were transferred, cycles in which double blastocysts were transferred, cycles in which preimplantation genetic diagnosis (PGD) was performed, cycles resulting in monozygotic twin pregnancies or ectopic pregnancies, and cycles in which pregnancy test results were not reached.

### 2.2. Ethical approval

This study was approved by the Sisli Memorial Hospital Ethical Committee (27.07.2022/62).

### 2.3. Ovarian stimulation protocol and oocyte retrieval

The controlled ovarian hyperstimulation was initiated on the second day of the menstrual cycle based on the individual body mass index (BMI), anti-Müllerian hormone level, basal antral follicle count and the history, if any, of previous response to gonadotropins. The initial estradiol, luteinizing hormone, and progesterone levels were determined using a chemiluminescence immunoassay (Abbott Architect Plus), and initial ultrasound examinations were done on the second day of the menstrual cycle. The stimulation protocols have been described in the previous research of our clinic.^[12]^ Ovarian stimulation was performed using gonadotropin-releasing hormone (GnRH) analogue suppression (short or long), GnRH antagonist protocol and recombinant follicle-stimulating hormone (Gonal-f; Merck, Switzerland) or a combination of recombinant follicle-stimulating hormone and recombinant luteinizing hormone (Pergoveris; Merck, Switzerland) or human menopausal gonadotropin (Ferring, Switzerland). Final oocyte maturation was triggered with either the injection of 250 mcg recombinant human chorionic gonadotropin (Ovitrelle; Merck, Switzerland) or GnRH analogue (Lucrin; Abbott Laboratories, USA).

Oocyte retrieval guided by transvaginal ultrasound was carried out 36 hours after triggering.

### 2.4. Intracytoplasmic sperm injection, blastocyst culture, and assessment

The cumulus cells were enzymatically removed (Hyarolunidase 80 IU/mL, Irvine Scientific, USA) 3 to 4 hours after oocyte retrieval, and oocytes were transferred to culture media (Life Global, Belgium). Oocytes were inseminated by intracytoplasmic sperm injection in a human tubal fluid medium (HTF; Life Global, Brussels, Belgium) with HEPES (Life Global, Brussels, Belgium) at × 400 magnification using Olympus IX70 and Olympus IX71 inverted microscopes. Embryos were cultured in single-step culture medium (Life Global, Brussels, Belgium). When embryos reached this stage on day 5 or 6, they were frozen using a Kitazato vitrification kit (Kitazato, Biopharma). Thawing was also performed using Kitazato vitrification medium, according to the manufacturer’s recommendations. Embryos were assessed for vitality and re-expansion at 30 minutes and 2 hours after thawing.

### 2.5. Endometrial preparation protocols

Endometrial preparation occurred during natural cycles for those with regular cycles, and artificial ones for those with irregular/anovulatory cycles. On the second day of menstruation, patients without uterine/ovarian issues were followed up for embryo transfer. When the follicle diameter was 16 to 20 mm, and luteinizing hormone level reached 15 IU/L and above, Ovitrelle (Ovitrelle, Merck Serono, Switzerland) triggered ovulation, and blastocyst transfer was performed 6 days after trigger. Crinone gel (Crinone 8%; Merck Serono, Switzerland) or Lutinus vaginal trophectoderm biopsy (TB) 2 × 100 mg (Lutinus 100 mg; Ferring, Germany) was used for luteal phase support. For the artificial cycle, the endometrium was prepared with a 2 mg Estradiol tablet (Estrofem, Novo Nordisk, Denmark) or a 3, 9 mg Estradiol patch (Climara, Bayer Turk, Turkey). Luteal phase support was initiated once the endometrium exceeded 7 mm after at least 12 days of estradiol valerate use as Crinone vaginal gel 2 × 1 or Lutinus 100 mg vaginal tablet 2 × 2. The β-hCG test was performed 9 days after blastocyst transfer. When this test was positive, a second β-hCG test was done 2 days later. The serum levels of hCG-β were determined using Chemiluminescence immunoassay (Abbott, Architect plus) with assay sensitivity of ≤ 1.2 m IU/mL.

### 2.6. Clinical outcomes

A positive pregnancy test result was defined as an initial β-hCG level ≥ 20 IU/L. According to the American Pregnancy Association guidelines on β-hCG levels at gestational weeks, β-hCG levels below 5 IU/L are considered negative for pregnancy, and levels above 25 IU/L are considered positive. A β-hCG level between 6 and 24 IU/L was considered a gray area. Based on this guideline, we considered an initial β-hCG level above 20 IU/L as a positive pregnancy test**.** Biochemical pregnancy is diagnosed only by detecting β-hCG in the serum, which does not develop into a clinical pregnancy. Clinical pregnancy loss was defined as loss occurring after ultrasonographic or clinical documentation of a fetus with a heartbeat. Live birth rate: Number of births resulting in at least 1 live-born baby expressed per 100 embryo transfer cycles^[13]^

### 2.7. Statistical analyses

Statistical Package for the Social Sciences version 21.0 (SPSS Inc., Chicago, IL) was used to analyze the data. To compare the groups for biochemical pregnancy loss, clinical pregnancy loss, and live birth, 1-way ANOVA for more than 2 group comparisons and a chi-square test for evaluating differences in frequencies between groups were performed. The results are presented in Table 1. Statistical significance was set at *P* < .05.

**Table 1:** Characteristics and cycle parameters of patients who became pregnant after fresh ET and frozen-thawed ET cycles.

	Fresh ET cycles (n = 738)				Frozen-thawed ET cycles (n = 2500)			
	Biochemical pregnancy loss (n = 75)	Clinical pregnancy loss (n = 98)	Live birth (n = 565)	*P* value	Biochemical pregnancy loss (n = 263)	Clinical pregnancy loss (n = 379)	Live birth (n = 1858)	*P* value
No. of cycles								
Female age (yr)	32.85 ± 4.53	33.00 ± 4.24	31.49 ± 4.13	.000†	31.05 ± 4.67	31.59 ± 4.46	30.77 ± 4.10	.002†
Paternal age (yr)	32.76 ± 4.34	32.97 ± 4.21	31.50 ± 4.10	.001†	34.84 ± 4.99	35.15 ± 5.27	34.32 ± 4.87	.006†
BMI (kg/m2)	24.22 ± 4.19	25.76 ± 5.43	24.20 ± 4.34	.006†	26.34 ± 5.06	25.85 ± 4.92	24.69 ± 4.47	.000†
AMH (ng/mL)	1.34 ± 1.38	1.60 ± 1.67	1.67 ± 1.57	.247†	4.42 ± 3.94	4.56 ± 3.51	4.09 ± 3.29	.030†
Duration of infertility (yr), n	3.71 ± 3.30	5.24 ± 4.66	4.14 ± 3.65	.130†	5.77 ± 3.89	6.08 ± 4.58	5.29 ± 3.83	.001†
Total number of previous cycles, n	3.01 ± 2.51	3.05 ± 2.46	1.51 ± 1.98	.000†	4.87 ± 2.19	4.94 ± 2.47	3.53 ± 1.82	.000†
Cause of infertility								
Male Factor	17.3% (13/75)	19.4% (19/98)	23.2% (131/565)	.407‡	32.3% (85/263)	31.1% (118/379)	36.3% (674/1858)	.098‡
Female Factor	52.0% (39/75)	43.9% (43/98)	38.1% (215/565)	.050‡	30.8% (81/263)	30.3% (115/379)	27.9% (518/1858	.436‡
Combined Factors	17.3% (13/75)	27.6% (27/98)	24.1% (136/565)	.285‡	15.6% (41/263)	19.5% (74/379)	14.1% (262/1858	.026‡
Unexplained infertility	13.3% (10/75)	9.2% (9/98)	14.7% (83/565)	.343‡	21.3% (56/263)	19.0% (72/379)	21.7% (404/1858)	.492‡
Serum ß-hCG (IU/L)								
İnitial ß-hCG	50.37 ± 24.31	114.86 ± 72.42	171.76 ± 109.64	.000†	68.41 ± 51.85	149.29 ± 96.99	193.57 ± 100.38	.000†
Second ß-hCG	65.39 ± 57.84	289.59 ± 201.44	441.42 ± 304.99	.000†	118.27 ± 140.97	390.34 ± 280.25	517.55 ± 287.42	.000†
Increase of initial and second ß-hCG (fold)	1.26 ± 0.94	2.51 ± 0.84	2.61 ± 0.77	.000†	1.49 ± 1.37	2.61 ± 0.90	2.67 ± 0.78	.000†

AMH = anti-müllerian hormone, BMI = body mass index, ET = embryo transfer, ß-hCG = beta-human chorionic gonadotropin.

* *P* < .05.

† One-way ANOVA.

‡ Chi-square test.

To determine the sensitivity and specificity of the threshold values for initial serum β-hCG levels, receiver operating characteristic (ROC) curve analysis was used. The threshold value, sensitivity, specificity, positive predictive value (PPV), negative predictive value (NPV), and area under the curve are given for both single fresh and frozen embryo transfers in Table 4. Regression analysis was used to analyze the effect of predictors on the initial serum β-hCG levels. The unstandardized coefficients (β values) and p-values are listed in Table 3. *P* values < .05 were considered effective for serum β-hCG levels.

**Table 2:** ß-hCG levels (IU/L) based on pregnancy outcomes from fresh or single frozen-thawed blastocyst transfer.

Pregnancy outcome	Increase rate of β-hCG levels(fold)	Fresh ET group (n = 738)	Frozen ET group (n = 2500)	*P* value	Initial β-hCG levels (IU/L)	Fresh ET group (n = 738)	Frozen ET group (n = 2500)	*P* value
Biochemical pregnancy loss	Mean SD	1.26 ± 0.94 n = 75	1.49 ± 1.37 n = 263	.158*	Mean SD	50.37 ± 24.31 n = 75	68.41 ± 51.85 n = 263	.000*
Clinical pregnancy loss	Mean SD	2.51 ± 0.84 n = 98	2.61 ± 0.90 n = 379	.345*	Mean SD	114.86 ± 72.42 n = 98	149.29 ± 96.99 n = 379	.000*
Live birth	Mean SD	2.61 ± 0.77 n = 565	2.67 ± 0.78 n = 1858	.211*	Mean SD	171.76 ± 109.64 n = 565	193.57 ± 100.38 n = 1858	.000*

ET = embryo transfer, IU/L = international unit/liter, SD = standard deviation, ß-hCG = beta-human chorionic gonadotropin.

* Student-*t* test.

**Table 3:** Multiple linear regression analysis.

Variables in multiple linear regression	Unstandardized coefficients	Standardized coefficients	t	*P* value	95% CI
Maternal age	−1.644	−0.066	−2.888	.004	−2.761 to 0.528
Paternal age	0.665	0.031	1.317	.188	−0.325 to 1.656
BMI	−4.496	−0.198	−11.091	.000	−5.291 to −3.702
Duration of infertility	−0.144	−0.005	−0.277	.782	−1.164 to 0.876
History of recurrent miscarriage	−8.976	−0.016	−0.910	.363	−28.318 to 10.367
Repeated implantation failure	−11.121	−0.028	−1.567	.117	−25.033 to 2.792

BMI = body mass index.

**Table 4:** Receiver operating characteristic curve analysis.

Variables	Fresh ET group (n = 738)			Frozen ET group (n = 2500)		
	Biochemical pregnancy loss	Clinical pregnancy loss	Live birth	Biochemical pregnancy loss	Clinical pregnancy loss	Live birth
Threshold value (IU/L)	83.5	96,5	116.5	98.5	119	131.5
Sensitivity (%)	0.88	0.63	0.82	0.80	0.71	0.71
Specificity (%)	0.82	0.62	0.70	0.81	0.62	0.68
PPV (%)	0.35	0.21	0.90	0.33	0. 20	0.87
NPV	0.98	0.92	0.54	0.97	0.93	0.50
AUC	0.93	0.64	0.81	0.89	0.59	0.76

AUC = area under the curve, ET = embryo transfer, NPV = negative predictive value, PPV = positive predictive value.

## 3. Results

The results of 3238 cycles, in which 738 single blastocyst transfers and 2500 frozen-thawed single embryo transfers with positive pregnancy tests were evaluated. The demographic and cycle characteristics of the cases in single fresh and frozen-thawed embryo transfer (FET) cycles according to the pregnancy results are shown in Table 1. Female and paternal ages were younger in the live birth group in both fresh and FET cycles, and BMI was lower in the live birth group in both fresh and FET cycles. Regardless of pregnancy outcome, the mean initial β-hCG levels in FET cycles were significantly higher than those in fresh cycles. In both fresh and FET cycles, β-hCG levels were higher in the live birth group than in the pregnancy loss group. The mean initial β-hCG value of the cases who reached live birth in fresh cycles was 171.76 ± 109.64 IU/L, which was significantly higher than the mean value of the group with biochemical pregnancy loss 50.37 ± 24.31 IU/L and the mean value of the group with clinical pregnancy loss 114.86 ± 72.42 IU/L (*P* < .001). In FET cycles, the mean initial β-hCG values (193.57 ± 100.38 IU/L) of the cases that reached live birth were statistically significantly higher than the biochemical group (68.41 ± 51.85 IU/L) and the clinical pregnancy loss group (149.29 ± 96.99 IU/L) (Table 1). The rate of increase in β-hCG did not differ between the fresh and FET cycles. However, the rate of increase of β-hCG level in both FET and fresh cycles was significantly lower in the group with biochemical pregnancy loss than in the group with live birth (Table 2).

Multivariable regression analysis showed that an increase in female age and BMI had a negative effect on β-hCG levels (Table 3).

**Figure 1.**
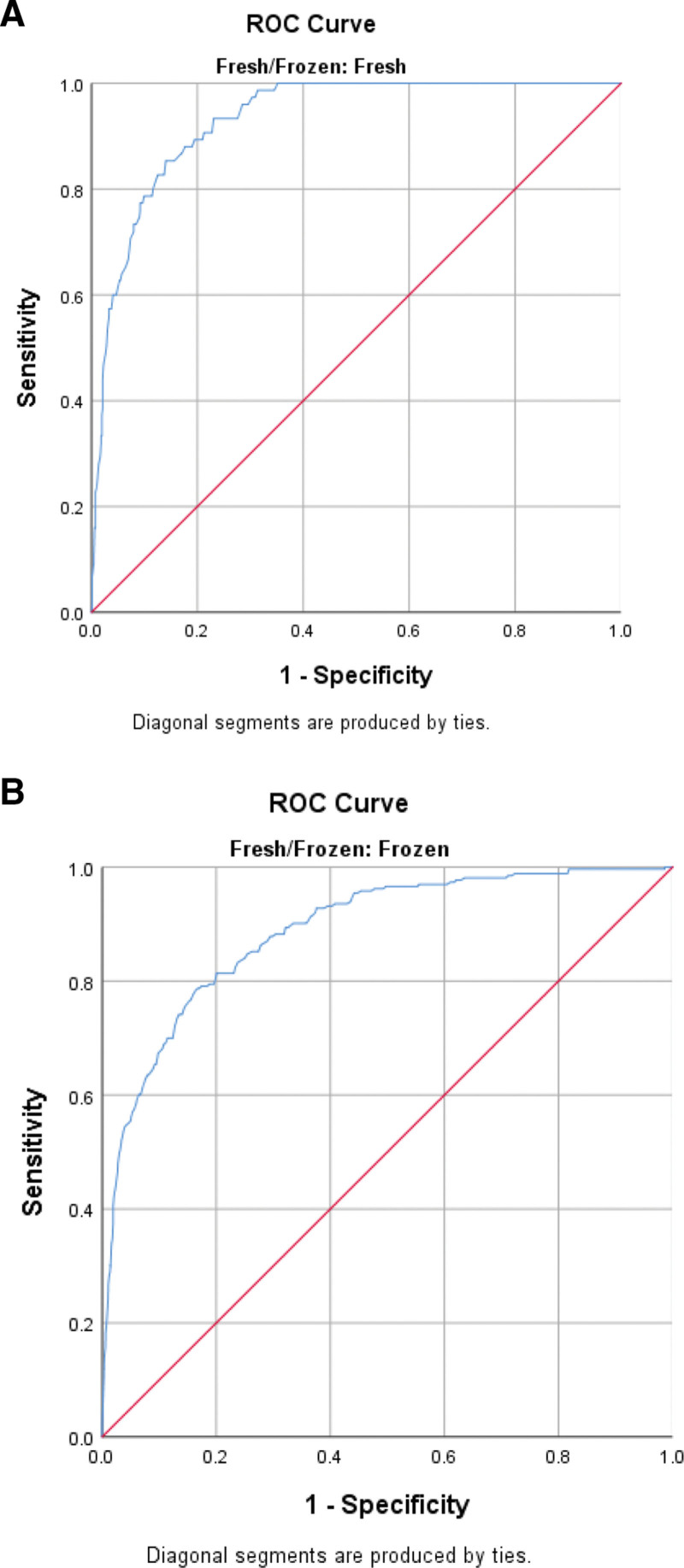
(A) Receiver operating characteristic (ROC) curve for serum hCG values for the prediction of biochemical pregnancy loss in fresh cycles. (B) Receiver operating characteristic (ROC) curve for serum hCG values for the prediction of biochemical pregnancy loss in FET cycles. FET = frozen-thawed embryo transfer, hCG = human chorionic gonadotropin.

**Figure 2.**
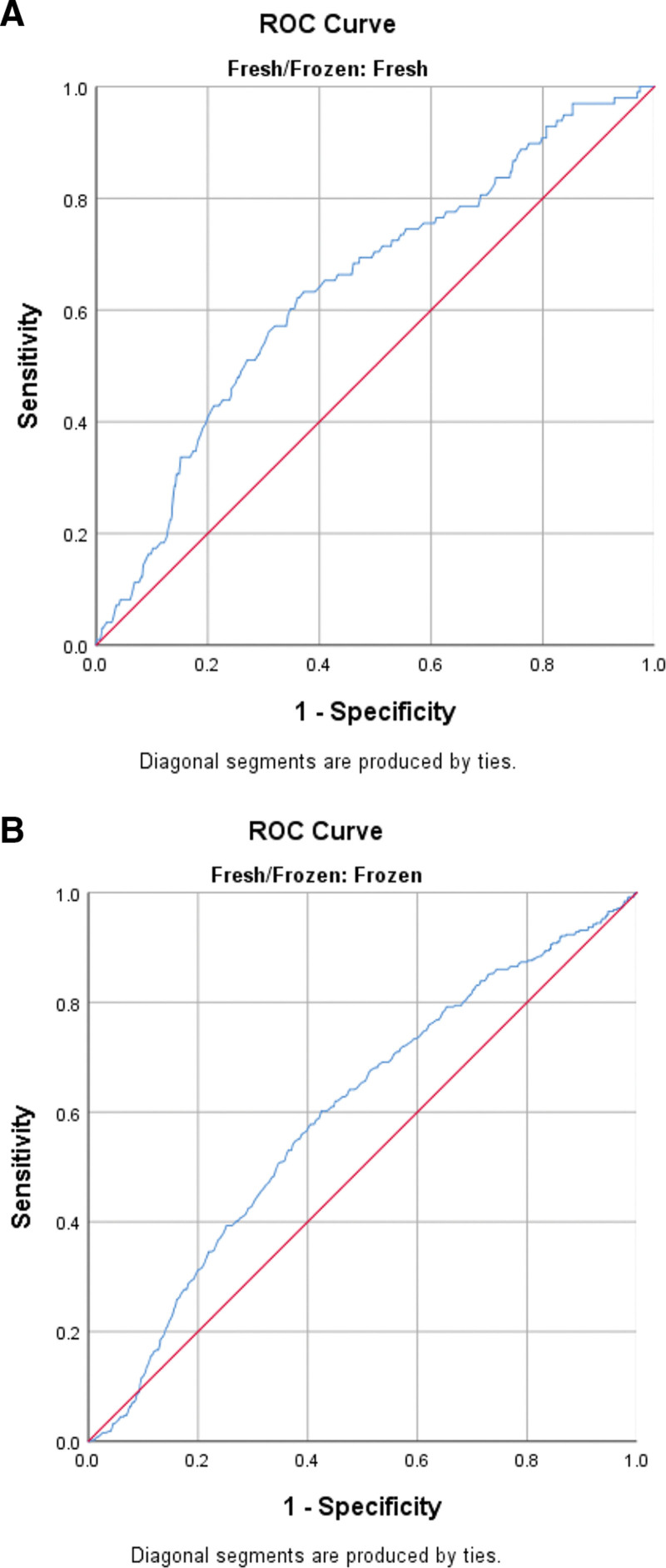
(A) Receiver operating characteristic (ROC) curve for serum hCG values for the prediction of clinical pregnancy loss in fresh cycles. (B) Receiver operating characteristic (ROC) curve for serum hCG values for the prediction of clinical pregnancy loss in FET cycles. FET = frozen-thawed embryo transfer, hCG = human chorionic gonadotropin.

**Figure 3.**
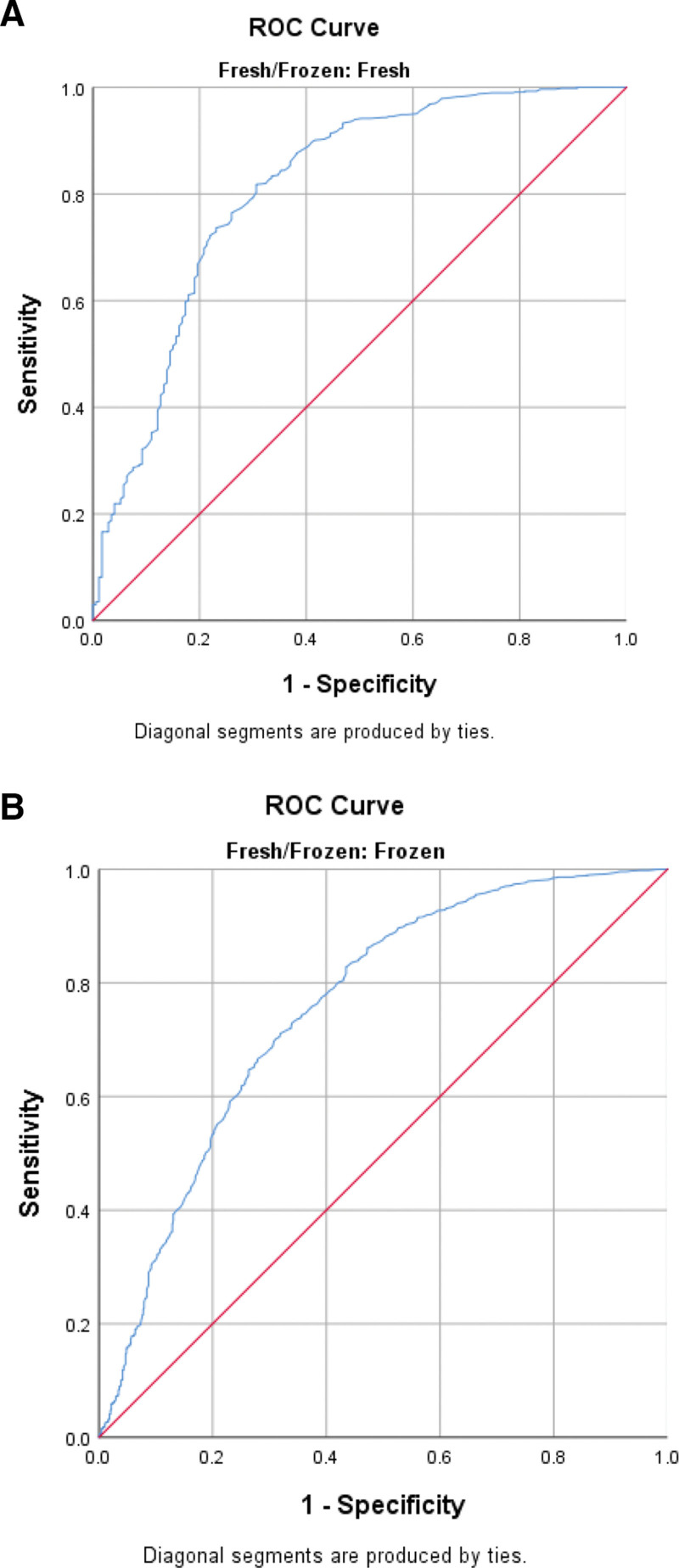
(A) Receiver operating characteristic (ROC) curve for serum hCG values for the prediction of live birth in fresh cycles. (B) Receiver operating characteristic (ROC) curve for serum hCG values for the prediction of live birth in FET cycles. FET = frozen-thawed embryo transfer, hCG = human chorionic gonadotropin.

ROC analysis was performed in fresh and frozen cycles to determine the threshold value of serum β-hCG according to pregnancy outcomes. The ROC curves for serum β-hCG values, shown in Figures 1, 2, and 3, predict biochemical pregnancy loss, clinical pregnancy loss, and live birth, respectively. This study determined that the threshold value for accurately predicting a successful live birth following fresh blastocyst embryo transfer was 116.5 IU/L. The sensitivity and specificity of this prediction were 80% and 70%, respectively. Additionally, the PPV was calculated to be 90%, whereas the NPV was 54%. The threshold value for predicting a live birth from frozen-thawed blastocyst transfer was 131.5 IU/L, with a sensitivity of 71%, specificity of 68%, PPV of 87%, and NPV of 50%.

The threshold value for predicting a clinical pregnancy loss from frozen-thawed blastocyst transfer was 119 IU/L, with a sensitivity of 71%, specificity of 62%, PPV of 20%, and NPV of 93%. The determined threshold value for the prediction of a clinical pregnancy loss resulting from a fresh blastocyst transfer was found to be 96.5 IU/L. This threshold value exhibited a sensitivity of 63%, specificity of 62%, PPV of 21%, and NPV of 92%.

The determined threshold value for the prediction of a biochemical pregnancy loss resulting from a fresh blastocyst transfer was found to be 83.5 IU/L. This threshold value exhibited a sensitivity of 88%, specificity of 82%, PPV of 35%, and NPV of 98%.

The established cutoff point for predicting biochemical pregnancy loss resulting from frozen-thawed blastocyst transfer was determined to be 98.5 IU/L. The sensitivity and specificity of this threshold were 80% and 81%, respectively, with a PPV of 33% and NPV of 97% (Table 4).

## 4. Discussion

In this study, we found that initial β-hCG levels and their rate of increase 2 days later effectively predicted pregnancy outcomes following blastocyst transfer in fresh and FET cycles. We also showed that initial β-hCG levels in FET cycles were significantly higher than those in fresh cycles, suggesting that freezing and thawing do not adversely affect embryos.^[14]^ Studies indicating that improved pregnancy results are linked to frozen-thawed embryo transfers also support this conclusion.^[15,16]^ Although there have been numerous studies on this topic, results regarding the day of β-hCG testing versus the day of embryo transfer are conflicting and unclear.^[7,9–11,17–19]^ Fertility centers may adhere to various procedures for initial β-hCG testing; however, the initial assessment of serum β-hCG levels is usually performed approximately 11 days after the transfer of cleavage-stage embryos or 9 days after blastocyst transfer. This timing corresponded to day 14 after oocyte retrieval. In our study, we performed β-hCG testing 9 days after a single blastocyst transfer. In fresh and frozen embryo transfer cycles, initial β-hCG levels were significantly higher in pregnancies resulting in live births than in biochemical and clinical pregnancy losses.

In contrast to our study, only 2 studies showed that the initial β-hCG levels were lower in FET cycles than in fresh cycles. In a smaller sample, Sites et al^[20]^ showed that the initial β-hCG levels were higher in fresh embryo transfers than in frozen embryo transfers. They suggested that this may be due to the apoptotic process in blastocysts caused by the slow freezing of embryos, which may lead to a decrease in β-hCG levels. In the same study, it was also reported that vitrification did not lead to a decrease in β-hCG levels. Similarly, Xue et al^[21]^ reported that slow freezing caused a decrease in β-hCG levels in day 3 embryos. In contrast to our study, these studies used day 3 embryo transfer.

Only a limited number of studies within the literature have indicated no significant difference in initial β-hCG values between fresh embryo transfer cycles and FET cycles. Lawler et al^[17]^ performed both day 3 embryo transfers and the transfer of multiple embryos. The elevated initial ß-hCG levels observed in cases of multiple pregnancies may have influenced the mean result. In a separate study by Reljič et al^[18]^, a comparison of hCG levels following blastocyst transfer demonstrated no difference in initial serum hCG levels between fresh and frozen cycles, regardless of pregnancy outcomes. Unlike our study, their research included both single- and multiple-blastocyst transfers. We intentionally excluded multiple embryo transfers in our study to prevent potential bias. Another recent study revealed that the initial β-hCG values did not differ between fresh and frozen embryo transfers. However, this study involved the evaluation of embryos with and without of PGD.^[9]^ Some studies have indicated that TB decreases initial β-hCG levels. Because all PGD embryos were frozen embryo transfers, the β-hCG value may have been lower in frozen embryo transfer cycles [19, 20]. In our study, we excluded PGD embryos in order to eliminate the effects of TB.

However, there are also studies in the literature that have achieved results similar to those of our study^[7,19]^ For example, a study by Oron et al^[7]^ showed that initial hCG levels were higher in FET cycles than in fresh cycles, as observed in our study. Nevertheless, unlike our results, they evaluated hCG test results on day 11 after blastocyst transfer but not on day 9. In addition, threshold values were given only for clinical pregnancies, and were 111 IU/L for predicting a clinical pregnancy for a fresh blastocyst and 137 IU/L for frozen-thawed blastocysts. Sung et al^[19]^ also showed that the β-hCG value was higher in frozen embryo transfers than in fresh cycles, and that the threshold values given 14 days after ovulation (9 days after blastocyst transfer) were higher in frozen cycles than in fresh cycles, regardless of pregnancy outcomes. They showed that the mean initial β-hCG values of cases reaching live birth in fresh cycles were 197.2 ± 98.1 IU/L and 222.6 ± 131.6I IU/L in frozen cycles. In our study, the mean initial β-hCG value of the cases reaching live birth was 171.76 ± 109.64 IU/L in fresh cycles and 193.57 ± 100.38 IU/L in frozen cycles.

Initial β-hCG levels can predict pregnancy outcomes, but the exact thresholds for prognosis are uncertain.^[7,17,19]^ Our study identified specific threshold values for serum β-hCG levels that can significantly improve the prediction of outcomes following blastocyst embryo transfer. It is important to tailor predictive models to a particular type of transfer (fresh or frozen-thawed) and intended outcome (live birth, clinical pregnancy loss, or biochemical pregnancy loss). The threshold values of 116.5 IU/L for fresh blastocyst transfers and 131.5 IU/L for frozen-thawed transfers have proven valuable in predicting successful live births, with varying levels of sensitivity and specificity.

Additionally, the thresholds of 83.5 IU/L and 98.5 IU/L are highly sensitive and specific for fresh and frozen-thawed transfers, respectively, in predicting biochemical pregnancy loss. However, it is critical to consider the clinical context and trade-offs between sensitivity and specificity when applying these thresholds in clinical practice. It is essential to conduct prospective validation studies in diverse patient populations further to refine the utility of biomarker thresholds in reproductive medicine. The threshold values given in the studies were different because the initial β-hCG values varied depending on the day of embryo transfer, whether PGD was performed, the sensitivity of the tests used by the clinics, and the day the test was performed. Our recommendation is that all clinics determine their own cutoff values.

In multivariate analysis, maternal age and BMI were found to influence β-hCG levels. An increase in maternal age and BMI was associated with lower β-hCG levels.^[6]^ Numerous studies have demonstrated a connection between elevated BMI and increased rates of pregnancy loss^[22,23]^ Although the reason for increased miscarriage rates at high BMI values is not clearly explained, some investigators have suggested a potential association with impaired endocrinological and/or biochemical conditions, whereas others have pointed out that this correlation may be linked to the reduced bioavailability of various hormones required for luteal support. The correlation between a decrease in β-hCG level and an increase in BMI appears to be linked to a higher pregnancy loss rate. Similarly, the negative effect of age on β-hCG levels also seems to correspond to an increase in pregnancy loss rates as female age increases.^[6]^

Our research was conducted at a single center under identical culture conditions and in the same laboratory for β-hCG testing. We eliminated all variables affecting the β-hCG value, excluding embryo transfer cycles by PGD and multiple embryo transfers. We believe that these are the strengths of the present study. A limitation of our study is that the number of patients in fresh embryo cycles was lower than that in FET cycles.

The results of this study confirmed that the initial levels of β-hCG and their rate of increase are crucial indicators for predicting pregnancy outcomes. When put into clinical use, especially during the early stages of pregnancy, these findings can serve as valuable tools for evaluating the risk of potential complications and for improving patient follow-up strategies. Additionally, these findings can guide the determination of the most effective treatment approach and create a strategic plan for early pregnancy follow-up. It is strongly recommended that β-hCG test values and their rate of increase be routinely employed in clinical practice to identify timely intervention in early high-risk pregnancies.

## Author contributions

**Conceptualization:** Gonul Ozer.

**Investigation:** Gonul Ozer.

**Methodology:** Gonul Ozer.

**Writing – original draft:** Gonul Ozer.

**Writing – review & editing:** Gonul Ozer.
